# Predictive role of blood-based indicators in neuromyelitis optica spectrum disorders

**DOI:** 10.3389/fnins.2023.1097490

**Published:** 2023-04-06

**Authors:** Xiqin Fang, Sujuan Sun, Tingting Yang, Xuewu Liu

**Affiliations:** ^1^Department of Neurology, Qilu Hospital, Shandong University, Jinan, China; ^2^Department of Neurology, Institute of Epilepsy, Shandong University, Jinan, China

**Keywords:** NMOSD, hemocyte, relapse, PLR, MLR

## Abstract

**Introduction:**

This study aimed to assess the predictive role of blood markers in neuromyelitis optica spectrum disorders (NMOSD).

**Methods:**

Data from patients with NMOSD, multiple sclerosis (MS), and healthy individuals were retrospectively collected in a 1:1:1 ratio. The expanded disability status scale (EDSS) score was used to assess the severity of the NMOSD upon admission. Receiver operating characteristic (ROC) curve analysis was used to distinguish NMOSD patients from healthy individuals, and active NMOSD from remitting NMOSD patients. Binary logistic regression analysis was used to evaluate risk factors that could be used to predict disease recurrence. Finally, Wilcoxon signed-rank test or matched-sample *t*-test was used to analyze the differences between the indicators in the remission and active phases in the same NMOSD patient.

**Results:**

Among the 54 NMOSD patients, neutrophil count, neutrophil-to-lymphocyte ratio (NLR), platelet-to-lymphocyte ratio (PLR), and systemic immune-inflammation index (SII) (platelet × NLR) were significantly higher than those of MS patients and healthy individuals and positively correlated with the EDSS score of NMOSD patients at admission. PLR can be used to simultaneously distinguish between NMOSD patients in the active and remission phase. Eleven (20.4%) of the 54 patients had recurrence within 12 months. We found that monocyte-to-lymphocyte ratio (MLR) (AUC = 0.76, cut-off value = 0.34) could effectively predict NMOSD recurrence. Binary logistic regression analysis showed that a higher MLR at first admission was the only risk factor for recurrence (*p* = 0.027; OR = 1.173; 95% CI = 1.018–1.351). In patients in the relapsing phase, no significant changes in monocyte and lymphocyte count was observed from the first admission, whereas patients in remission had significantly higher levels than when they were first admitted.

**Conclusion:**

High PLR is a characteristic marker of active NMOSD, while high MLR is a risk factor for disease recurrence. These inexpensive indicators should be widely used in the diagnosis, prognosis, and judgment of treatment efficacy in NMOSD.

## 1. Introduction

Neuromyelitis optica spectrum disorder (NMOSD) is a severe idiopathic inflammatory demyelinating disease that usually affects the optic nerve, spinal cord, and some lesions may even involve the brain (Wingerchuk et al., 2015; Shosha et al., 2018). In the past, NMOSD was often confused with multiple sclerosis (MS). However, with the discovery of aquaporin-4 (AQP-4) antibodies and the expansion of clinical features, neuroimaging, and cerebrospinal fluid (CSF) feature, the two diseases were distinguished (Lennon et al., 2004). NMOSD leads to more serious neurological dysfunction as the disease develops, an increase in the number of recurrences, and eventually serious visual or motor damage (Jarius et al., 2012; Houzen et al., 2017). Therefore, it is important to identify factors that can predict NMOSD recurrence and assist in early diagnosis. Previous studies have found that indicators in serum or CSF can predict the recurrence of NMOSD. However, the process of testing for most indicators is expensive and complex. Therefore, it is necessary to identify indicators with higher economic benefits.

Gong et al. (2020) found that promoting neutrophil apoptosis can alleviate brain damage in NMOSD mice. Platelets have been found to drive inflammation in experimental autoimmune encephalomyelitis (EAE) models and infiltrate the central nervous system (CNS) early in the disease (D’Souza et al., 2018; Orian et al., 2021). In addition, recent studies have also found that neutrophil-to-lymphocyte ratio (NLR), platelet-to-lymphocyte ratio (PLR), monocyte-to-lymphocyte ratio (MLR), monocyte × NLR (systemic inflammation response index, SIRI), platelet × NLR (systemic immune inflammation index, SII) have been found to be markers of inflammation and can predict the prognosis of a number of tumors (Chen et al., 2021; Li et al., 2021, 2022; Ginesu et al., 2022). In addition to predicting the prognosis of a variety of tumors, the appeal indicators have recently been used to predict other diseases. NLR, PLR and MLR were found to be associated with disease activity in polymyalgia rheumatica (Jung et al., 2019). SII and SIRI were also significantly higher in patients with schizophrenia and bipolar disorder than in normal people (Wei et al., 2022). During the COVID-19 epidemic, NLR, PLR, MLR, SII and SIRI all effectively predicted death (Ghobadi et al., 2022). Remarkably, NLR, MLR, PLR, and SII have also been found to be significantly higher in MS patients than in healthy people (Gokce et al., 2023).

These indicators are mainly composed of four hemocytes: platelets, neutrophils, lymphocytes, and monocytes. The role of these hemocytes in the pathogenesis of NMOSD is poorly studied, but is well documented in the pathogenesis of MS. Previous studies have shown that platelets are involved in the inflammatory process (Morrell et al., 2014). At the same time, platelets express and secrete adhesion molecules that accumulate in inflamed tissues and stimulate the adhesion of platelets to white blood cells. The result of this adhesion is the formation of platelet-white blood cell aggregates leading to further inflammation (Yan et al., 2014). In addition, glucocorticoid-induced tumor necrosis factor receptor family-related protein ligand (GITRL) is expressed during the differentiation of megakaryocytes into platelets, and when GITRL binds to GITR, it will activate the mitogen-activated protein kinases (MAPK) signaling pathway and aggravate the inflammation (Wang et al., 2021). These findings suggest that platelets may be a pro-inflammatory blood component.

Neutrophils can disrupt the blood–brain barrier (BBB) (Pierson et al., 2018). After the destruction of the BBB, pro-inflammatory factors in peripheral circulation and secreted by neutrophils can enter the CNS and promote inflammation. In addition, neutrophils release neutrophilic extracellular traps (NETS) after death, and the production of NETS may also maintain CNS autoimmunity (Pierson et al., 2018). Correspondingly, increased infiltration of neutrophils in the CNS has been observed in MS patients and EAE models (Rumble et al., 2015). Together, these results suggest that an increase in neutrophils may contribute to autoimmune inflammation in CNS.

Normally, the presence of the BBB prevents peripheral monocytes from entering the CNS. However, when inflammation occurs, the BBB is destroyed and monocytes from the peripheral circulation can enter the CNS. After entering the CNS, the derived subsets of monocytes not only can directly mediate the inflammatory response, but also can aggravate autoimmune inflammation by presenting autoantigens (Spiteri et al., 2022). Similarly, monocyte enrichment was found to correlate with onset and severity of EAE (King et al., 2009). Thus, like neutrophils, the elevation of peripheral monocytes may contribute to the development of CNS autoimmune diseases, including NMOSD.

Unlike peripheral inflammation, lymphocytes play a key role in CNS autoimmune diseases. Balasa et al. (2021) confirmed that in MS patients, lymphocytes are activated peripherally, and then cross the BBB, trigger an immune response, and further self-sustain, leading to myelin and axon damage. This seems to imply that an increase in lymphocytes contributes to the occurrence and maintenance of central autoimmune responses. However, multiple studies have found that the lymphocytes in people with MS are not significantly higher than in healthy people, and may even be lower (Lim et al., 2016; Akaishi et al., 2021). The possible explanation for this is that during the onset of MS, peripheral lymphocytes migrate to the CNS through the injured BBB, but the hematopoietic system cannot compensate for the loss of peripheral lymphocytes (Zhou et al., 2022). As a result, peripheral lymphocytes tend to be normal or even decreased, while lymphocytes in the CNS tend to increase. This is also a unique feature of lymphocytes in CNS autoimmune diseases. Based on this, the reduction of peripheral lymphocytes may not indicate a reduction of central autoimmune response.

Based on the distinct functions of these four hemocytes, we speculated that the values of all the five indicators composed of these four hemocytes might be proportional to the severity of the immune response seen in NMOSD.

In addition to hemocyte counts, lipid and glucose levels in the blood are also readily available test indicators. Among the many blood lipid indexes, high-density lipoprotein (HDL) is one of the most important. HDL has a number of benefits, including anti-inflammatory and antioxidant functions (Nazir et al., 2020). As an important carrier of HDL in the process of cholesterol transport, apolipoprotein A1 (ApoA1) has been found to be significantly reduced in patients with NMOSD (Zhong et al., 2013), suggesting that HDL may incur similar changes in NMOSD. The triglyceride-glucose index (TyG) is widely used to assess insulin resistance (Tahapary et al., 2022). A study by Maghbooli et al. (2021) linked insulin resistance with NMOSD, indicating that higher insulin resistance is a risk factor for NMOSD. Therefore, we speculated that TyG would also be significantly implicated in NMOSD.

In conclusion, we designed this trial to identify promising indicators of NMOSD that are convenient and inexpensive to detect, and are capable of predicting the likelihood of recurrence of disease activity and overall prognosis of patients.

## 2. Methods

### 2.1. Participants

Data were retrospectively collected from patients admitted to the Qilu Hospital of Shandong University, from January 2017 to September 2021,. All patients were in the acute phase at admission and were diagnosed with NMOSD at discharge according to the 2015 International Panel for NMO Diagnosis (IPND) criteria. Patients had not received any immunosuppressive or steroid therapy prior to admission. They showed no symptoms of any other systemic infection or diseases during the 14 days prior to the blood sample collection. There was no combination of hematological and autoimmune diseases in the other systems. After enrollment in the study, patients were excluded if they met the following criteria: (1) lost to follow-up, (2) unable to receive ongoing care, as requested by neurologists, and (3) during follow-up, a blood disorder or autoimmune disease of another system or any other condition requiring additional steroids or immunotherapy developed.

### 2.2. Information collection

Fasting blood samples were collected from patients after admission, and relevant laboratory tests were performed to collect several indicators, including white blood cells, specifically neutrophils, monocytes, and lymphocytes, platelets, HDL, and triglycerides. The expanded disability status scale (EDSS) score, assigned by a specialist neurologist, was used to assess the patient’s disability status. To detect demyelinating antibodies and paraneoplastic antibodies, the cerebrospinal fluid of NMOSD patients was obtained using lumbar puncture. Blood samples from patients with MS (MS group) and age-and sex-matched healthy individuals (HC group) were collected in a 1:1 ratio as controls. All MS patients that were included were in the acute phase, and patients with clinically isolated syndrome were excluded. Each patient with NMOSD was followed for 12 months. All patients and healthy individuals agreed to participate in the study and gave written informed consent.

### 2.3. Clinical definition

Based on the clinical presentation of patients on admission, the following categories were identified: optic neuritis (ON), acute transverse myelitis (ATM), area postrema syndrome (APS), brainstem syndrome (BSS), diencephalic syndrome (DS) and cerebral syndrome (CS). Relapse was defined as new or worsening neurological symptoms lasting more than 24 h without other causes. New neurological findings and/or new lesions were confirmed by magnetic resonance imaging (MRI) (Khalilidehkordi et al., 2020).

### 2.4. Statistical analysis

Differences in categorical variables among the three groups were analyzed using the chi-square test or Fisher’s exact test. The Shapiro–Wilk test was used to determine whether continuous variables conformed to a normal distribution. The independent sample *t*-test was used for continuous variables conforming to a normal distribution, and the Mann–Whitney U test was used for continuous variables that did not conform to a normal distribution. The Shapiro–Wilk test was also used to determine whether paired continuous variables were normally distributed. The matched sample *t*-test was used for analysis when both paired variables were in line with normal distribution; otherwise, the Wilcoxon signed-rank test was used for analysis. Receiver operating characteristic (ROC) curve analysis was used to evaluate the predictive ability of each index. Spearman’s correlation coefficient was used to determine the factors associated with EDSS scores. Binary logistic regression analysis was used to determine the risk of factors affecting disease recurrence, and a multicollinearity diagnostic model was used to screen for appropriate variables. All statistical analyzes were performed using Statistical Package of Social Sciences (SPSS) version 26.

## 3. Results

### 3.1. Baseline characteristics

A total of 54 patients with NMOSD were included in the analysis, most of which were female (*n* = 44, 81.5%). The average age was 46.57 (SD: 15.62). The mean EDSS score was 4.05 (SD: 2.70). The most common clinical manifestation was ATM (*n* = 30, 55.6%). Followed by APS (*n* = 5, 9.3%), ON + ATM (*n* = 5, 9.3%), BSS (*n* = 4, 7.4%), ON + BSS (*n* = 3, 5.3%), ATM + BSS (*n* = 2, 3.7%), ON (*n* = 2, 3.7%), CS (*n* = 1, 1.9%), CS + DS (*n* = 1, 1.9%), DS + BSS (*n* = 1, 1.9%). Eleven patients (20.4%) relapsed within 12 months of discharge. Medication and relapse information of patients with NMOSD and MS are shown in Supplementary Table S2.

### 3.2. Differences in hemocyte and metabolic indexes between three groups

Univariate analysis showed that neutrophil count, NLR, PLR, and SII in the NMOSD group were significantly higher than those in the HC and MS groups. In addition, the platelet count and SIRI in the NMOSD group were significantly higher than those in the HC group, while they did not show a significant difference between the NMOSD and MS groups (Table 1). Among the six indicators that were significantly higher in the NMOSD group than in the HC group, ROC curve analysis showed that SII had the highest sensitivity at the cut-off value (cut-off value = 450.81, sensitivity = 74.1%), and PLR had the highest specificity under the cut-off value (cut-off value = 288.98, specificity = 100%; Supplementary Figure S1; Supplementary Table S1).

**Table 1:** Comparison of blood indexes between the three groups.

Variables	NMOSD (*n* = 54)	MS (*n* = 54)	HC (*n* = 54)	*P*1	*P*2	*P*3
Female (*n*%)	44 (81.5)	43 (79.6)	38 (70.4)	0.500	0.130	0.187
Age (SD)	46.57 (15.62)	37.72 (12.84)	48.26 (9.81)	0.002*	0.907	<0.001*
NEU (SD)	4.96 (2.37)	4.14 (1.91)	3.01(1.10)	0.040*	<0.001*	<0.001*
LYM (SD)	1.67(0.85)	2.02 (0.74)	1.76 (0.50)	0.007*	0.241	0.061
MON (SD)	0.37 (0.21)	0.39 (0.16)	0.39 (0.10)	0.606	0.204	0.271
PLT (SD)	269.63 (71.75)	254.06 (66.89)	241.35 (52.40)	0.231	0.028*	0.252
NLR (SD)	4.10 (3.56)	2.25(1.37)	1.82 (0.79)	0.005*	<0.001*	0.136
MLR (SD)	0.23 (0.13)	0.20 (0.08)	0.23 (0.08)	0.068	0.745	0.001*
PLR (SD)	215.50 (144.06)	141.34 (70.53)	147.90 (52.35)	0.005*	0.049*	0.223
SIRI (SD)	1.12(0.78)	0.87 (0.65)	0.73 (0.48)	0.109	0.004*	0.191
SII (SD)	1144.00 (1064.14)	583.24 (503.02)	448.38 (231.04)	0.003*	<0.001*	0.060
HDL (SD)	1.23(0.34)	1.36 (0.30)	1.34 (0.30)	0.039*	0.176	0.672
TyG (SD)	8.40 (0.54)	8.25 (0.47)	8.33 (0.58)	0.184	0.545	0.443
Median EDSS (Q1,Q3)	3 (2,7)	–	–	–	–	–
Antibody						
AQP4	40	–	–	–	–	–
MOG	3	–	–	–	–	–
AQP4 + MOG	2	–	–	–	–	–
AQP4 + para- neoplastic	1	–	–	–	–	–
None	8	–	–	–	–	–

NEU: neutrophil, LYM: lymphocyte, MON: monocyte, PLT: platelet.

* P1: Comparison results between NMOSD group and MS group, P2: comparison results between NMOSD group and HC group, P3: comparison results between MS group and HC group.

### 3.3. Factors associated with severity of NMOSD

Based on our results comparing the NMOSD group with the HC group, we further analyzed the association between hemocyte-related indicators and EDSS scores in NMOSD patients. Spearman’s correlation analysis showed that neutrophil, NLR, PLR, SIRI and SII were positively correlated with EDSS score (Table 2).

**Table 2:** Spearman’s correlation analysis between hemocyte indexes and EDSS.

Variables	*p* value	Correlation coefficient
NEU	001	0.423
PLT	0.096	0.229
NLR	001	0.446
PLR	015	0.350
SIRI	004	0.389
SII	<0.001	0.467

NEU: neutrophil, PLT: platelet.

### 3.4. Factors predicting recurrence in patients with NMOSD

**Figure 1:**
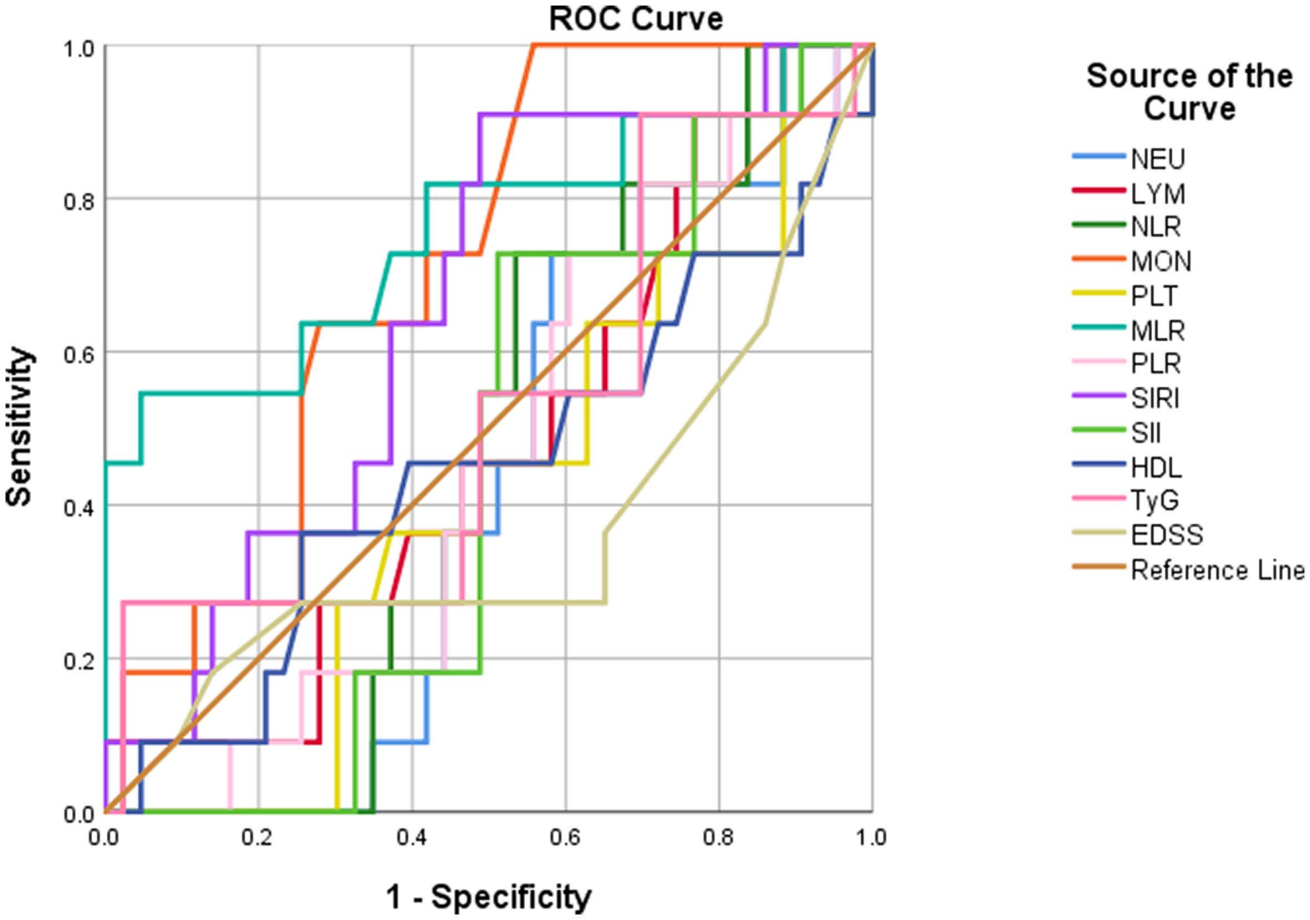
ROC curve analysis for predicting recurrence.

**Figure 2:**
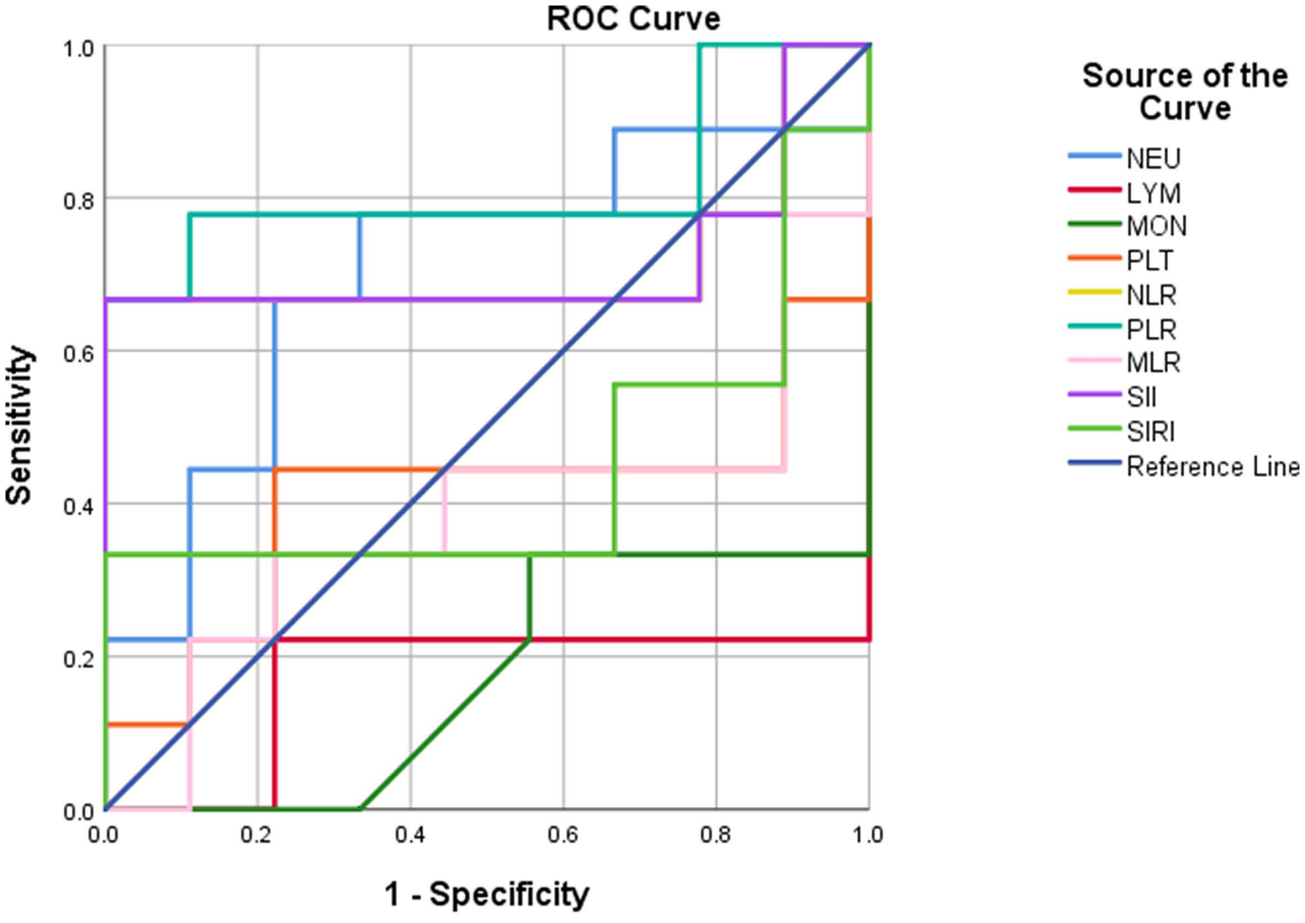
ROC curve analysis for distinguishing active phase from remission phase.

To explore the factors associated with recurrence in patients with NMOSD, we analyzed the differences in indicators at admission between the 11 patients with recurrence and the remaining 43 patients. ROC curve analysis of the admission test results of the 54 patients showed that MLR (AUC = 0.76, 95% CI = 0.576–0.944, cut-off value = 0.34, sensitivity = 54.5%, specificity = 95.3%) and monocyte count (AUC = 0.71, 95% CI = 0.565–0.856, cut-off value = 0.305, sensitivity = 100%, specificity = 44.2%) were effective predictors of recurrence (Table 3; Figure 1). After excluding collinearity factors and after adjusting for other factors, binary logistic regression analysis showed that the risk of recurrence increased by approximately 18% for every 0.01 unit increase in MLR at first admission (*p* = 0.027, OR = 1.173, 95% CI = 1.018–1.351; Table 4; Figure 2).

**Table 3:** ROC curve analysis for predicting recurrence.

Variables	AUC	*P* value	95% CI
NEU	0.419	0.408	0.260–0.577
LYM	0.478	0.822	0.297–0.659
MON	0.710	0.033	0.565–0.856
NLR	0.463	0.707	0.307–0.619
PLT	0.409	0.356	0.233–0.586
MLR	0.760	0.008	0.576–0.944
PLR	0.457	0.660	0.288–0.626
SIRI	0.658	0.110	0.492–0.823
SII	0.448	0.599	0.290–0.607
HDL	0.450	0.614	0.244–0.656
TyG	0.520	0.838	0.316–0.724
EDSS	0.366	0.173	0.157–0.575

NEU: neutrophil, LYM: lymphocyte, MON: monocyte, PLT: platelet.

**Table 4:** Binary logistic regression analysis for risk factors for recurrence.

Variables	OR	95% CI	*P* value
Age	0.956	0.876–1.043	0.311
Male	5.021	0.128–196.963	0.389
NEU	0.926	0.491–1.746	0.812
PLT	0.995	0.976–1.015	0.616
MON	0.977	0.001–867.978	0.995
MLR	1.173	1.018–1.351	0.027
HDL	8.117	0.122–542.090	0.329
TyG	1.951	0.181–20.998	0.581
EDSS	0.694	0.433–1.112	0.129
Treatment			0.768
Steroid hormone	Ref	–	–
Steroid hormone+MMF	0.426	0.038–4.759	0.488
Steroid hormone+AZA	2.727	0.114–65.513	0.536
Rituximab	–	–	0.999

NEU: neutrophil, PLT: platelet, MON: monocyte, MMF: mycophenolate mofetil, AZA: azathioprine.

### 3.5. Different responses to treatment in relapsing and remitting NMOSD patients

We collected the hemocyte test results of 9 patients in active relapse and 9 patients in remission during the follow-up period, while the other 36 patients were excluded as they received treatment or were re-checked in local hospitals. All the 18 patients were required to be more than 6 months following initial discharge and on continuous therapy when the blood results were reviewed. Similarly, ROC curve analysis was performed on various indicators during follow-up to identify indicators that could distinguish patients in the relapsed active stage from those in remission (Table 5). The results showed that the lymphocyte and monocyte count of patients in the relapsed active stage were significantly lower than those in the remission stage at follow-up, with an AUC of 0.827 (cut-off value = 1.365, sensitivity = 100%, specificity = 77.8%) and 0.827 (cut-off value = 0.43, sensitivity = 100%, specificity = 66.7%), respectively. In contrast, PLR was higher in the relapsed active stage (AUC = 0.815; cut-off value = 169.99, sensitivity = 77.8%, specificity = 88.9%). Furthermore, the Wilcoxon signed-rank test or matched sample *t*-test showed that 9 patients in the relapsed active phase had no significant difference in the three indicators at follow-up compared with the first admission, while the 9 patients in the remission phase showed significant increases in lymphocytes and monocytes, and a decrease in PLR after treatment (Table 6). Further analysis showed that after treatment, lymphocyte, monocyte, and PLR were not significantly affected by any regimen (Supplementary Table S4). Specific medication regimens for these 18 patients are shown in Supplementary Table S3.

**Table 5:** ROC curve analysis for distinguishing active phase from remission phase.

Variables	AUC	P value	95% CI
NEU	0.703	0.145	449–959
LYM	0.173	0.019	0–0.396
MON	0.173	0.019	0–0.370
PLT	0.407	0.508	0.117–0.698
NLR	0.716	0.122	0.441–0.991
MLR	0.383	0.402	0.105–0.660
PLR	0.815	0.024	0.592–1.000
SIRI	0.444	0.691	0.150–0.739
SII	0.716	0.122	0.441–0.991

NEU: neutrophil, LYM: lymphocyte, MON: monocyte, PLT: platelet.

**Table 6:** Comparison of pre- and post-treatment indicators between NMOSD patients with and without relapse.

Paired variables	Relapse (*n* = 9)	Without relapse (*n* = 9)
NEU	0.038	0.605
LYM	0.359	0.039
MON	0.359	0.013
PLT	0.672	0.178
NLR	0.038	0.093
MLR	0.478	0.066
PLR	0.659	0.017
SIRI	0.594	0.109
SII	0.139	0.081

NEU: neutrophil, LYM: lymphocyte, MON: monocyte, PLT: platelet.

## 4. Discussion

This study focused on the significance of hemocytes and their derived markers in NMOSD. Our results indicate that PLR and MLR are two important indices in NMOSD whereby PLR is a specific indicator for patients with active NMOSD, while MLR can predict disease recurrence.

Orian et al. (2021) found that thrombocytopenia prevented EAE in mice, thus it is clear that platelets play a role in driving inflammation in EAE (D’Souza et al., 2018). Although studies have been largely limited to MS, or experimental models of MS, NMOSD and MS are both inherently idiopathic inflammatory demyelinating diseases. This suggests that changes that occur in MS, may also occur in NMOSD. Our results confirmed that platelet counts were indeed higher in NMOSD patients than in healthy individuals. However, no more significance of platelet elevation was found. Instead, our results showed another more significant marker. The PLR of patients with active NMOSD was not only higher than that of the HC and MS groups, but also significantly higher than that of NMOSD patients in remission, which means that NMOSD patients with high PLR do not receive effective treatment. Additionally, PLR positively correlated with EDSS score, indicating that PLR not only plays a role in diagnosis but also allows for a simple initial assessment of the severity of NMOSD.

Prior to PLR, NLR was also found to be significantly higher in NMOSD patients than in the healthy population, which was further confirmed in our study (Benetou et al., 2020; Zhou et al., 2021). Similar to the study of Zhou et al. (2021), we also found an association between NLR and EDSS score. However, the NLR of NMOSD patients in both the active and remission phases was higher than that of healthy individuals. As Contentti et al. (2021) stated, NLR cannot strictly represent the active phase of the disease. Nevertheless, NLR is still an important indicator because no significant changes in NLR were observed even in patients with MS, suggesting that NLR can also be used as a potential differentiator between MS and NMOSD. The high levels of NLR in NMOSD patients may be due to the high levels of neutrophils, because the lymphocyte count in NMOSD patients was not significantly altered compared with the HC group. The increase in neutrophils is not surprising, as promoting neutrophil apoptosis can reduce brain damage in NMOSD mice (Gong et al., 2020), suggesting that there is an abnormal increase in neutrophils in NMOSD patients. Two studies by Saadoun et al. (2012) and Liu et al. (2020) also confirmed that neutrophils are involved in the pathogenesis of NMOSD.

SII has recently been extensively studied by oncologists and has been recognized as a prognostic marker for a variety of tumors. In our study, SII was also observed to be significantly higher in the NMOSD group than in the HC and MS groups and positively correlated with EDSS score. However, as with NLR, it was not significantly different between patients in active and remission, nor was it associated with the risk of recurrence. The abnormal elevation of NLR and SII may indicate a medical history of NMOSD, and their further significance remains to be explored.

Although HDL has anti-inflammatory and antioxidant functions, Li et al. (2010) also found that HDL in patients with NMOSD was significantly different from that in healthy people. However, in our study, no potential significance of HDL in NMOSD was found. Zhong et al. (2013) found that ApoA1 was significantly decreased in patients with NMOSD, and Li et al. (2010) also found that the ratio of ApoB to ApoA1 was significantly increased in patients with NMOSD and was proportional to the severity of disease. Both studies suggest that ApoA1 may be a protective factor during NMOSD. However, since only a small percentage of our patients were tested for apolipoproteins, we were unable to further verify the significance of apolipoprotein in NMOSD. TyG has recently been widely studied in the field of coronary heart disease, and it has also been found to be associated with poor prognosis in neurological diseases such as acute ischemic stroke and Alzheimer’s disease (Lee et al., 2021; Gentreau et al., 2022). Although Maghbooli et al. (2021) also found that high insulin resistance was a risk factor for NMOSD, our study did not find abnormal TyG in patients with NMOSD, which may suggest that the TyG index could not accurately represent the degree of insulin resistance in NMOSD.

In addition, we found that MLR could effectively predict NMOSD recurrence. This means that NMOSD patients with high MLR should prolong first-line therapy or receive second-line therapy earlier to prevent recurrence. Wang et al. (2022) found that MLR was associated with the prognosis of acute ischemic stroke and speculated that MLR might be related to the level of inflammation. However, our results did not find significant changes in MLR levels in patients with NMOSD, suggesting abnormalities in the function, but not the number, of monocytes and lymphocytes in active NMOSD. In addition, the lymphocyte and monocyte count of patients in the remission phase were significantly higher than when they had entered the active phase. This indicates that an increase in lymphocytes and monocytes may determine the effectiveness of treatment. However, this finding is surprising because the administration of steroid hormones reduces monocytes and lymphocytes, and most of the patients in our study were treated with steroid hormones. Kong et al. (2017) found that a proportion of CD14^+^CD16^++^ monocytes, a non-classical monocyte subset, were significantly increased in NMOSD patients, and was closely related to the upregulation of interleukin 6 (IL-6) and other proinflammatory mediators. Accordingly, we speculated that after effective treatment, the increase in monocytes may be due to the proliferation of the anti-inflammatory subset exceeding the suppression of the proinflammatory subset. Surprisingly, an increase in monocyte count was accompanied by an increase in lymphocyte count. Since the release of AQP-4 antibody is mainly related to B cells (Nie and Blair, 2022), research and treatment of NMOSD has mainly focused on targeting B cells. A study by Yang et al. (2021) comprehensively assessed changes in peripheral blood lymphocyte subsets in patients with NMOSD. They found significantly higher levels of CD19^+^ B cells during active NMOSD than in healthy people. However, they also found significant reductions in the CD4^+^T cell/CD8^+^T cell ratio and NK cell levels, indicating distinct properties of T and NK cells than B cells in NMOSD. Therefore, the increase of lymphocytes may be due to an increase in some lymphocyte inflammatory subsets. The use of some drugs may also cause an increase in lymphocytes as well as monocytes, and due to the small sample size, we cannot completely rule out the effect of drugs. Therefore, whether the increase in monocytes and lymphocytes means the treatment is effective needs further research. Finally, more attention should be paid to the pathological changes of NMOSD as the decline of NK cells was also shown to be absent in MS (Chen et al., 2022).

Our study had some limitations. First, the sample size of this retrospective study was small, which was not only reflected in the overall sample number, but also in the number of patients who relapsed. This may have prevented us from identifying the underlying significance of certain indicators. Second, the 12-month follow-up period was a limitation. Finally, we did not examine monocyte and lymphocyte subset counts in our patients, which hinders further interpretation of our results.

In conclusion, this study demonstrated the significance of two blood-based predictive measures, PLR and MLR, in NMOSD. High PLR is a specific marker of NMOSD activity and can be used in judgment of treatment efficacy or diagnosis, whereas emphasis on MLR can help reduce recurrence rates because high MLR is closely related to recurrence. Testing for these two indicators is straightforward, inexpensive, and highly accepted by patients, and thus, should be widely used in NMOSD.

## Data availability statement

The original contributions presented in the study are included in the article/Supplementary material, further inquiries can be directed to the corresponding author.

## Ethics statement

The study was approved by the ethics committee of Qilu Hospital, Shandong University (2020065). Written informed consent to participate in this study was provided by the participants’ legal guardian/next of kin, for the publication of any potentially identifiable images or data included in this article.

## Author contributions

XL and XF had the idea for the study, responsible for study design, responsible for data analysis, data interpretation, writing, and reviewing of the manuscript. XF, SS, and TY were responsible for data collection. All authors contributed to the article and approved the submitted version.

## Funding

This study was supported by the National Natural Science Foundation of China (grant no. 81873786).

## Conflict of interest

The authors declare that the research was conducted in the absence of any commercial or financial relationships that could be construed as a potential conflict of interest.

## Publisher’s note

All claims expressed in this article are solely those of the authors and do not necessarily represent those of their affiliated organizations, or those of the publisher, the editors and the reviewers. Any product that may be evaluated in this article, or claim that may be made by its manufacturer, is not guaranteed or endorsed by the publisher.
