# Supplementary material for: Predictive role of blood-based indicators in neuromyelitis optica spectrum disorders
Source: Front Neurosci. 2023 Apr 6;17:1097490. doi: 10.3389/fnins.2023.1097490 (PMC10115963; doi:10.3389/fnins.2023.1097490)

Table S1 ROC curve analysis for distinguishing NMOSD group from HC group

Variables	AUC	P value	95% CI	Cut off	sensitivity	specificity
NEU	0.824	0.001	0.721-0.887	3.97	59.3%	90.7%
PLT	0.622	0.028	0.517-0.728	242	70.4%	51.9%
NLR	0.725	0.001	0.630-0.820	2.14	63.0%	72.2%
PLR	0.610	0.049	0.502-0.717	288.98	25.9%	100%
SIRI	0.659	0.004	0.556-0.763	1.09	40.7%	88.9%
SII	0.741	<0.001	0.649-0.834	450.81	74.1%	64.8%

Abbreviations: NEU:neutrophil, PLT:platelet

Table S2 Treatment regimen and condition of NMOSD and MS patients

	NMOSD group (n=54)	MS group (n=54)
Therapeutic regimen[a] (n%)		
Steroid hormone	20 (37.0)	-
Steroid hormone+MMF	26 (48.1)	-
Steroid hormone+AZA	5 (9.3)	-
Rituximab (n%)	3 (5.6)	-
Fingolimod (n%)	-	22 (40.7)
Terifluramide (n%)	-	29 (53.7)
Dimethyl fumarate (n%)	-	3 (5.6)
Mean interval from the first discharge to the first relapse of NMOSD, month (SD)[b]	6.36 (3.33)	-
Mean interval from the last relapse to the current inclusion of MS, month (SD)	-	9.94 (4.14)
Classification of MS (n%)		
RR	-	45 (83.3)
SP	-	3 (5.6)
PP	-	1(1.9)
PR	-	1(1.9)
Unkonwn	-	4 (7.4)

Abbreviations: MMF: mycophenolate mofetil, AZA: azathioprine. RR: relapsing remitting type; SP: secondary progressive type; PP: primary progressive type; PR: progressive relapsing type.

[a]: It mens that all medication regiments refer to continuing treatment regiments after discharge, and all NMOSD and MS patients were treated with a steroid pulse therapy upon admission for acute episodes.

[b]: It means that this interval is the mean interval of the 11 patients who relapsed because only 11 patients with NMOSD relapsed during the follow-up period.

Table S3 Treatment regimen of NMOSD patients with and without relapse

Relapse (n=9)			Without relapse (n=9)	
Patient No.	Therapeutic regimen	Interval from the first discharge to the first	Patient No.	Therapeutic regimen

		relapse, month		
Patient 1	Steroid hormone	11	Patient 1	Steroid hormone+MMF
Patient 2	Steroid hormone+MMF	4	Patient 2	Steroid hormone+MMF
Patient 3	Steroid hormone+MMF	7	Patient 3	Steroid hormone
Patient 4	Steroid hormone	3	Patient 4	Steroid hormone
Patient 5	Steroid hormone+MMF	2	Patient 5	Steroid hormone+MMF
Patient 6	Steroid hormone	6	Patient 6	Steroid hormone
Patient 7	Steroid hormone	8	Patient 7	Steroid hormone+MMF
Patient 8	Steroid hormone	9	Patient 8	Steroid hormone
Patient 9	Steroid hormone+AZA	4	Patient 9	Steroid hormone+MMF

Abbreviations: MMF: mycophenolate mofetil, AZA: azathioprine.

Table S4 Differences in hematologic indexes before and after treatment in NMOSD patients treated with different regiments.

Paired variables	Steroid hormone (n=9)	Steroid hormone +MMF (n=8)	Steroid hormone +AZA (n=1)
LYM (P value)	0.331	0.239	-
MON (P value)	0.313	0.275	-
PLR (P value)	0.251	0.338	-

Abbreviations: LYM: lymphocyte, MON:monocyte, AZA: azathioprine, MMF: mycophenolate mofetil.

Figure S1 ROC curve analysis for distinguishing NMOSD group from HC group

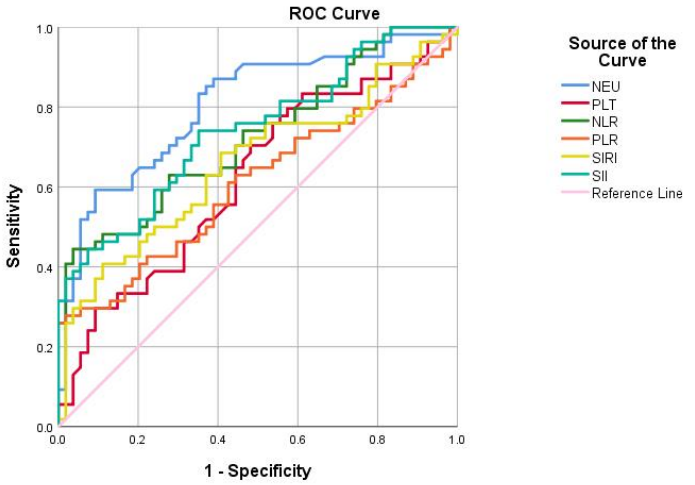

Supplement: Supplementary file 1 [file Data_Sheet_1.PDF]
